# Whole-genome homozygosity mapping reveals candidate regions affecting bull fertility in US Holstein cattle

**DOI:** 10.1186/s12864-020-6758-y

**Published:** 2020-05-04

**Authors:** Juan Pablo Nani, Francisco Peñagaricano

**Affiliations:** 1 Department of Animal Sciences, University of Florida, 2250 Shealy Drive, Gainesville, FL 32611 USA; 2 Estación Experimental Agropecuaria Rafaela, Instituto Nacional de Tecnología Agropecuaria, 22-2300 Rafaela, SF Argentina; 3 University of Florida Genetics Institute, University of Florida, Gainesville, FL 32610 USA

**Keywords:** Inbreeding depression, Male fertility, Runs of homozygosity, Sire conception rate

## Abstract

**Background:**

Achieving rapid genetic progress while maintaining adequate genetic diversity is one of the main challenges facing the dairy industry. The increase in inbreeding can be used to monitor the loss of genetic diversity. Inbreeding tends to increase the proportion of homozygous loci, some of which cause homozygosity of recessive alleles that results in reduced performance. This phenomenon is known as inbreeding depression and tends to be most prominent on fitness-related traits, such as male fertility. Traditionally, inbreeding has been monitored using pedigree information, or more recently, genomic data. Alternatively, it can be quantified using runs of homozygosity (ROH), i.e., contiguous lengths of homozygous genotypes observed in an individual’s chromosome.

**Results:**

The objective of this study was to evaluate the association between ROH and sire conception rate. ROH were evaluated using 268 k genetic markers in 11,790 US Holstein bulls. Interestingly, either the sum, mean, or maximum length of ROH were negatively associated with bull fertility. The association analysis between ROH and sire fertility was performed comparing 300 high-fertility vs. 300 low-fertility bulls. Both the average and sum of ROH length were higher in the low-fertility group. The enrichment of ROH regions in bulls with low fertility was assessed using a Fisher’s exact test. Nine regions were significantly enriched in low-fertility compared to high-fertility bulls. Notably, these regions harbor genes that are closely related to sperm biology and male fertility, including genes exclusively or highly expressed in testis.

**Conclusions:**

The results of this study can help not only to manage inbreeding in genomic selection programs by designing custom mating schemes, but also to better understand the mechanisms underlying male fertility in dairy cattle.

## Background

Inbreeding is the mating of individuals that share at least one common ancestor, and therefore, making the number of direct ancestors in the pedigree to be smaller than expected. Inbreeding cannot be avoided in populations of limited size. In addition, in livestock species, only a limited number of parents are used to produce the offspring for the next generation. In dairy cattle, the extensive use of semen from elite bulls and common breeding practices such as mating half-brother and half-sister or first or second cousins, has led to a decrease in effective population size along with a steady increase in inbreeding coefficients for the last decades [1].

Increased levels of inbreeding leads to inbreeding depression, which is the reduction of the mean phenotypic value for any trait under selection [2]. Inbreeding depression has been found to be especially detrimental for fitness-related traits, traits that are mostly under non-additive regulation [3]. Several studies have shown the negative impact of inbreeding on fertility and fertility-related traits in dairy cattle, such as bull semen quality [4], early embryo development [5], number of inseminations per conception and calving ease [6], calving interval and age at first calving [7], nonreturn to service and calving rate [8], dystocia and stillbirths [9].

Inbreeding depression can arise from three basic mechanisms: (i) the partial dominance hypothesis refers to the expression of deleterious recessive alleles in homozygous individuals and therefore expressing the deleterious genotype; (ii) the over-dominance hypothesis where the heterozygous alleles expressing the superior genotype are underrepresented in a population with increased inbreeding; and (iii) the epistasis hypothesis where a combination of heterozygous alleles expressing a superior genotype is less frequent as inbreeding arises [10]. In general, inbreeding depression is calculated by regressing the individual phenotype on its own pedigree inbreeding coefficient [11], assuming that the inbreeding depression is a linear function of the inbreeding level of the individual. However, this method relies mainly on pedigree information, which is prone to have missing information and relative high error rates. Also, pedigree inbreeding is based on the expected proportion of the genome that is identical by descent but the true genomic relationship between two individuals deviate as a consequence of Mendelian sampling, leading to underestimated values for inbreeding coefficients [12, 13].

As high-throughput genotyping became available, different methods using genome-wide molecular information were developed to estimate the realized proportion of the genome shared by two individuals. This information can be used to calculate inbreeding levels even for animals with no pedigree information [10, 14]. It should be noted that these methods do not allow to distinguish between identical by descent (IBD) from identical by state (IBS), driving the inbreeding coefficient to be overestimated. In addition, two individuals with exactly the same genomic inbreeding could have different inbred regions in the genome, and hence, the methods are unable to discriminate different levels of local inbreeding, which may affect different traits.

High density single nucleotide polymorphism (SNP) arrays allow detecting long IBS segments within an individual’s chromosome. These continuous homozygous segments assumed to be inherited from a recent common ancestor are called runs of homozygosity (ROH) and provide a potential solution for many of the problems mentioned with the pedigree and genomic methods. Indeed, ROH has been proven to be the most powerful method to calculate inbreeding coefficients [15]. The use of ROH is gaining ground in dairy cattle genomics, such as for mapping regions of high homozygosity and their impact on inbreeding depression [16], for revealing regions under strong selection [17] and also, for detecting variants associated with female [18] and male fertility [19]. The main objective of this study was to evaluate the association between ROH and sire conception rate, the US national phenotypic evaluation of dairy bull fertility.

## Results

### Assessment of runs of homozygosity

**Fig. 1:**
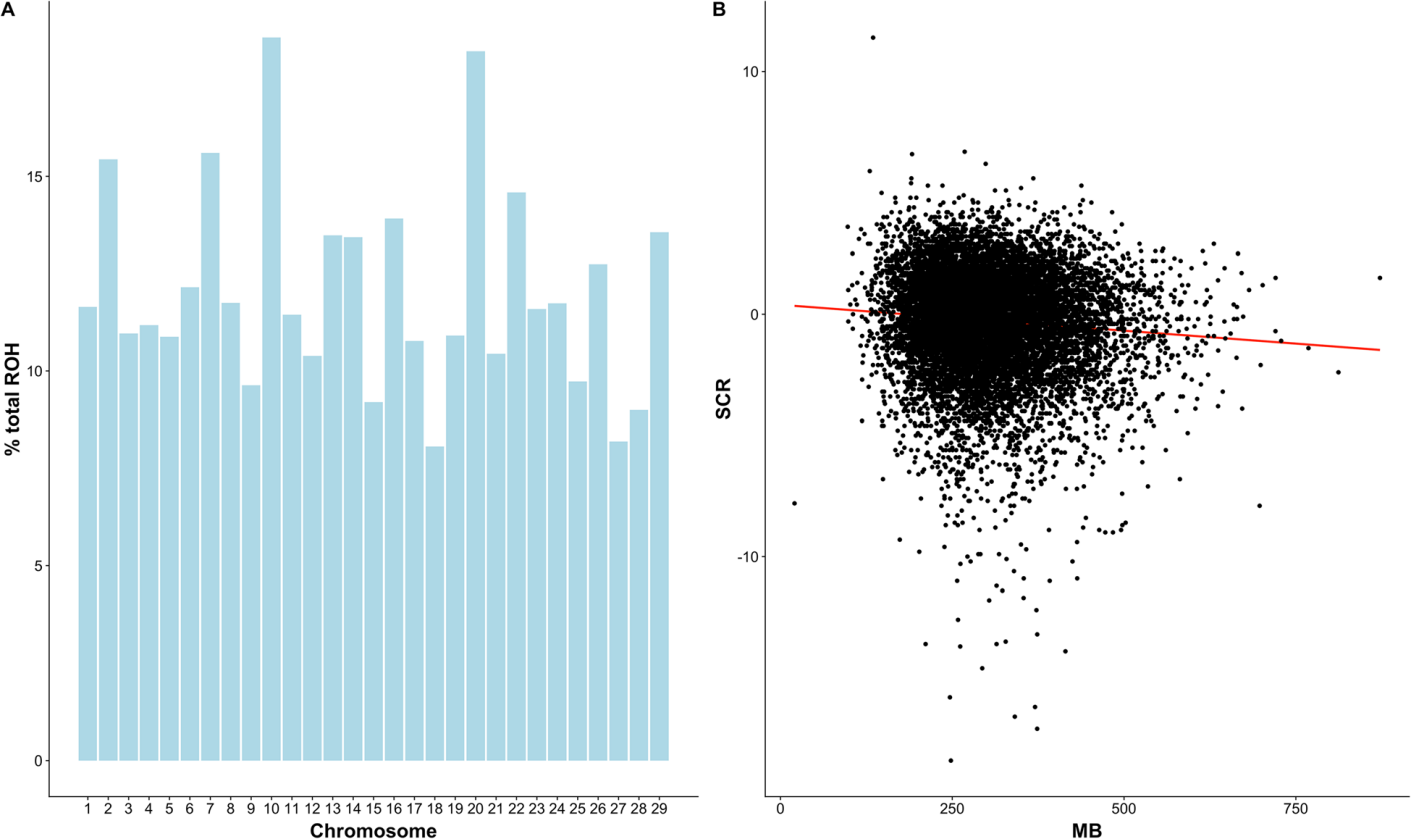
Assessment of runs of homozygosity in the entire US Holstein bull population. **a**: Average percentage of total homozygosity (Y axis) for each chromosome (X axis). **b**: Total homozygosity, calculated as the sum of runs of homozygosity (Y axis) versus sire conception rate (SCR), a measured of dairy bull fertility (X axis)

A total of 692,131 runs of homozygosity (ROH) were found in the entire US Holstein bull population (*n* = 11,790). The mean of ROH length was 5187 kilobases, equivalent to 543 consecutive homozygous SNP. The maximum length of ROH was 140,731 kilobases, equivalent to 13,417 SNP. The number of ROH segments per bull ranged from 13 to 104, with an average of 58.7. The percentage of homozygous regions for each chromosome is shown in Fig. 1a. Chromosomes BTA2, BTA7, BTA10 and BTA20 presented higher degree of homozygosity than the rest of the autosomes, with more than 15% homozygosity. The least homozygous chromosomes were BTA18 and BTA27 with around 8% homozygosity. The average homozygosity for the autosomal genome was 10.41%.

### Association between ROH and dairy bull fertility

Association between homozygosity and male fertility was assessed by plotting the total homozygous regions for each animal (sum of ROH) versus sire conception rate records (Fig. 1b). The red line represents the regression of sire conception rate on ROH, which clearly indicates a negative association between bull fertility and the amount of homozygosity (regression coefficient *β* =  − 0.002, *t*-value = −9.3, *P*-value ≤ 0.01). The Pearson’s correlation coefficient between SCR and sum of ROH was equal to −0.09, while the correlation between ROH and pedigree inbreeding was equal to 0.74. The relationship between SCR and ROH was also evaluated for each individual autosome, and the same trend was observed for each chromosome (data not shown).

**Fig. 2:**
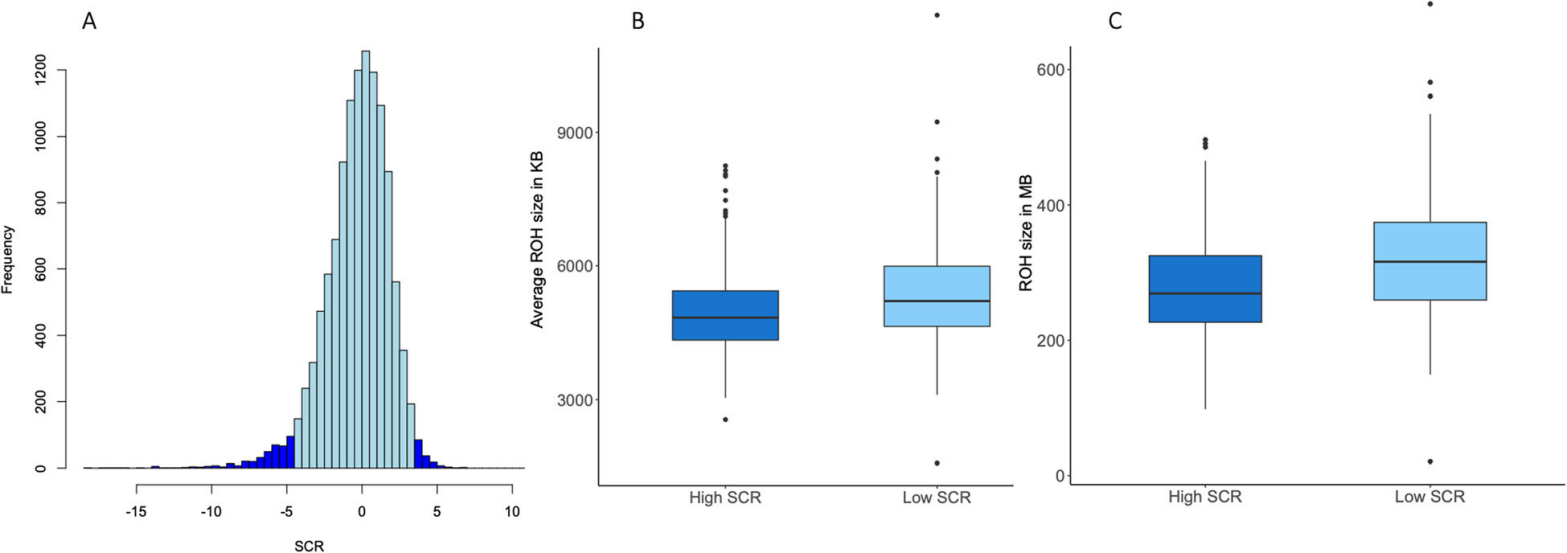
Association between runs of homozygosity and dairy bull fertility. **a**: Histogram showing the distribution of sire conception rate (SCR) records for the entire US Holstein bull population (*n* = 11,790). The top 300 high-fertility bulls and the bottom 300 low-fertility bulls are highlighted in blue. **b**: Distribution of average length of runs of homozygosity for high-fertility (*n* = 300) compared to low-fertility (*n* = 300) bulls. **c**: Distribution of total homozygosity, calculated as the sum of all runs of homozygosity, for high-fertility (*n* = 300) compared to low-fertility (*n* = 300) bulls

Differences in ROH metrics were evaluated between 300 high-fertility and 300 low-fertility bulls (Fig. 2a). Interestingly, the average size of ROH was significantly different (*P*-value ≤ 0.01) between fertility groups. Mean values of ROH were 4950 kb and 5360 kb for high-fertility and low-fertility bulls, respectively (Fig. 2b). The total homozygosity content was measured as the sum of all ROH segments for each animal, and significant differences (*P*-value ≤ 0.01) were found between groups. Indeed, the mean of total ROH length was 323 Mb for low-fertility bulls and 280 Mb for high-fertility bulls (Fig. 2c).

### Enrichment of ROH in low-fertility bulls

**Fig. 3:**
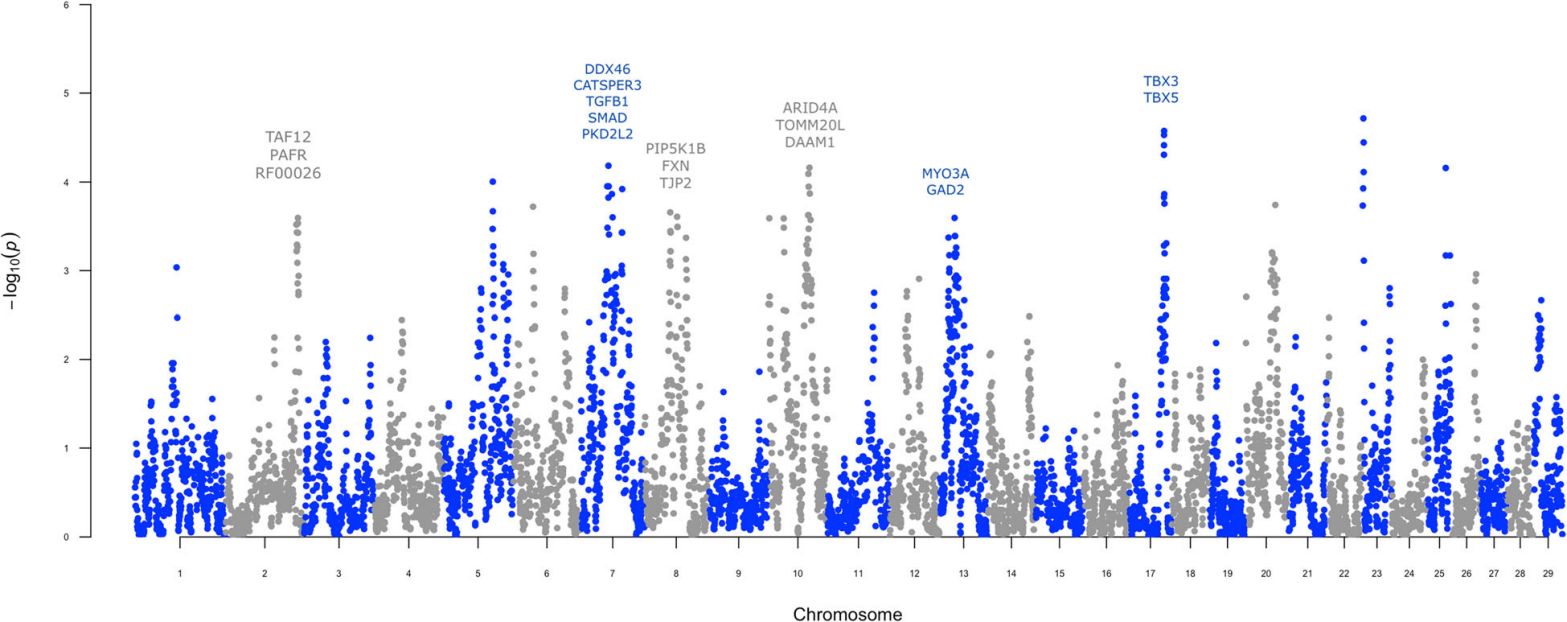
Whole-genome homozygosity mapping. The enrichment of runs of homozygosity (ROH) in low-fertility bulls was evaluated using a Fisher’s exact test. This Manhattan plot shows the significance of each ROH region (Y axis) across the entire autosome genome (X axis). Putative genes affecting bull fertility are highlighted

Genomic regions where ROH overlapped across animals were identified using the entire US Holstein bull population. A total of 6758 overlapping ROH were detected across the genome (Additional file 1). The mean length of overlapping ROH was 96.3 kb, containing an average of 12 SNP. The longest overlapping region was 1342 kb containing 65 SNP. All overlapping regions were mapped to the latest bovine refence genome (ARS-UCD1.2). The enrichment of overlapping ROH segments in animals with low fertility was evaluated using a Fisher’s exact test, comparing the top 300 high-fertility against the bottom 300 low-fertility Holstein bulls. Sixty ROH regions were found to be significantly enriched in low-fertility bulls after Bonferroni correction (adjusted *P*-value ≤ 0.05, Fig. 3). These overlapping ROH segments were filtered and only those containing at least five SNP were considered. As a result, 9 genomic regions over 9 different chromosomes were retained for further analysis (Table 1, Additional file 2).

**Table 1:** Homozygous regions significantly enriched in low-fertility Holstein bulls

Chr.	Start (bp)	End (bp)	***P***-value	t-value	Candidate Genes
**2**	122,242,657	125,425,072	2.5 × 10^− 04^	− 0.57	TAF12, PAFR, RF00026
**5**	85,191,780	85,569,862	9.9 × 10^− 05^	−6.18	–
**7**	46,103,009	49,396,693	6.5 × 10^−05^	−7.68	DDX46, CATSPER3, TGFB1, SMAD, PKD2L2
**8**	44,817,928	45,351,612	2.2 × 10^−04^	−8.56	PIP5K1B, FXN, TJP2
**10**	68,307,915	71,956,178	8.1 × 10^−05^	−2.28	ARID4A, TOMM20L, DAAM1
**13**	26,260,323	27,088,377	4.1 × 10^−04^	−10.74	MYO3A, GAD2
**17**	59,986,109	60,771,002	2.7 × 10^−05^	−14.23	TBX3, TBX5
**23**	2,352,780	4,018,158	1.9 × 10^−05^	−3.16	–
**25**	32,215,384	32,240,080	6.9 × 10^−05^	−10.74	–

### Validation of significant ROH

The significant association between these nine ROH segments and dairy bull fertility was validated using the entire US Holstein bull population. From the original 9 overlapping ROH regions, eight of them were significantly associated with sire conception rate records in the whole population (|t-value| ≥ 2), with the exception of the region located in chromosome 2 that showed a *t*-value equal to −0.57. The position of the ROH segments along with *P*-values (Fisher’s exact test) and the *t*-values (validation analysis) can be found in Table 1. Interestingly, strong candidate genes were found in almost every region. In fact, genes directly related with male fertility, i.e., genes with strong evidence of playing an important role in testis development, spermatogenesis, or sperm function, most of them exclusively or highly expressed in testis, were found in the ROH regions located in chromosomes 2, 7, 8, 10, 13 and 17. These candidate genes are also listed in Table 1.

## Discussion

Inbreeding is a growing concern for the dairy cattle industry. Traditionally, inbreeding has been assessed using either pedigree or genomic relationships, but these methods are not perfect, mainly because their inability to explore specific regions of high homozygosity in the genome. The impact of inbreeding and increased homozygosity on fitness traits, such as longevity and fertility, has been studied in dairy cows, but the impact on dairy bull fertility has received less attention, despite the fact that one sire is generally used to inseminate hundreds or even thousands of cows. To the best of our knowledge, this is the first study investigating how local inbreeding affects dairy bull fertility. Our goal was to investigate how specific homozygous regions of the genome impact male fertility in dairy cattle and reveal the putative candidate genes located in these regions. First, we evaluated the association between runs of homozygosity (ROH) and sire conception rate (SCR) records using 11,790 Holstein bulls with 268 k SNP markers. Second, we separated the entire population into 300 high-fertility and 300 low-fertility bulls in order to investigate the potential enrichment of ROH segments in subfertile sires, i.e., bulls with very low SCR values. Finally, we mapped the significant ROH regions to the bovine genome in order to identify candidate genes that may affect male fertility in cattle.

### Assessment of runs of homozygosity

Runs of homozygosity were detected in all the bulls evaluated in this study. The average length of ROH was longer than 5 Mb, and hence, it can be defined as a long ROH. Long ROH are likely to arise from a recent common ancestor, less than 10 generations ago [20]. The average ROH per animal, *n* ≈ 59, was higher than the average reported by Kim and collaborators [17] (*n* ≈ 40), probably because we used a 100 SNP window threshold rather than the 50 SNP window, although very similar results were found for total autozygosity (~ 10%). The distribution of total homozygosity among chromosomes was relatively even, but higher in chromosomes 2, 7, 10 and 20. The distribution of ROH is correlated with genomic features such as GC content, but also selection and recombination rate, with longer ROH occurring more often in low recombination areas of the genome [21, 22]. Similarly, Kim and collaborators [17] found that the distribution of ROH was more variable across the genomes of selected animals. They also found several regions with higher homozygosity, supporting the hypothesis that frequency and size of ROH can be affected by artificial selection.

### Association between ROH and dairy bull fertility

The impact of homozygosity on service sire fertility was assessed by calculating the relationship between total ROH and SCR values. The correlation was consistently negative for all autosomes. These results are in agreement with the premise that bull fertility is a complex phenotype which is influenced by several features in the genome with relatively small effects. Runs of homozygosity has been previously used to study the negative impact of local homozygosity in dairy cow fertility, including increased intervals from first to last insemination [18] and embryo survival [23]. The effect of homozygosity on sperm biology has also been evaluated, and ROH regions affecting total number of spermatozoa and percentage of live spermatozoa were previously reported [19]. It should be noted that none of these ROH regions overlap with our findings.

### Enrichment of ROH in low-fertility bulls

The 300 top and 300 bottom Holstein bulls in the SCR distribution clearly represent highly fertile and subfertile sires, respectively. Notably, there was a clear difference in the levels of homozygosity between these two fertility groups, in terms of average length of ROH and also in terms of total homozygosity, represented as the sum of ROH. Moreover, although none of the identified ROH regions were exclusive to low-fertility bulls, nine ROH segments were found to be significantly enriched in the subfertile bulls. Importantly, eight out of the nine ROH regions were statistically validated in the entire population, and therefore, these results are not false positives due to population structure. It is also worth noting that some of the identified regions are located near previously reported regions associated with dairy bull fertility, either regions with additive effects [24] or non-additive effects [25]. Altogether, these results suggest that homozygosity might be an important risk factor for bull subfertility, and the accumulation of multiple recessive variants might be a relevant component of the genetic architecture underlying sire conception rate in dairy cattle.

### Significant regions and candidate genes

The region in BTA2 harbors three strong candidate genes directly implicated in male fertility, namely *TAF12*, *PAFR*, and *RF00026*. Gene *TAF12* encodes the TATA-box binding protein associated factor 12, which regulates a testis-specific gene expression program in primary spermatocytes required for terminal differentiation of male germ cells [26]. The platelet activating factor (PAF) has a direct effect on sperm biology by affecting the motility, capacitation, acrosome reaction and fertility of spermatozoa in several species [27]. Gene *RF00026* encodes the U6 spliceosomal RNA and homologs of this gene has been associated with testicular hypoplasia in Nellore cattle [28] and also found in homozygous regions affecting male fertility in humans.

The region in BTA7 contains several candidate genes, including *DDX46*, *CATSPER3*, *TGFB1*, *SMAD*, and *PKD2L2*. Gene *DDX46* encodes a member of the DEAD box protein family and is implicated in a number of cellular processes involving alteration of RNA secondary structure. Interestingly, *DDX46* is highly expressed in testis where it plays a critical role in the final step of the first meiotic prophase in male germ cells [29]. Gene *CATSPER3* is exclusively expressed in the testis and encodes a sperm-specific ion channel. This gene has been associated with sperm function and male fertility in several species including cattle [30]. Abnormalities in the CATSPER gene family have been associated with idiopathic male infertility with normal semen parameters in humans [31]. Both genes *TGFB1* and *SMAD5* are clustered together in BTA7 and encode ligands that play important roles in the regulation of cell development and growth [32]. Interestingly, both ligands impact testis development during the fetal period [33] and abnormal function of these genes is associated with human non-obstructive azoospermia [34]. Finally, gene *PKD2L2* is also located in this significant region of BTA7, and although its role in sperm biology and male fertility is not well understood, it is highly expressed in mammalian testis [35].

The significant ROH segment in BTA8 harbors at least 3 putative genes related to male fertility, namely *PIP5K1B*, *FXN*, and *TJP2*. Gene *PIP5K1B* along with *PIP5K1A* play an important role in spermatogenesis in mice, since knockout males lacking both proteins exhibit complete infertility due to decreased sperm number and a 25% reduction in testes weight compared to wild-type males [36]. Gene *FXN* encodes a mitochondrial protein that regulates mitochondrial iron transport and respiration. Interestingly, a knockout mutation in *FXN* produces embryonic lethality in mice [37]. Gene *TJP2* encodes a tight junction protein that is critical for the blood-testis barrier, and its deficiency has been associated with reduced fertility and pathological changes in the testis [38].

The region in BTA10 contains three strong candidate genes, *ARID4A*, *TOMM20L*, and *DAAM1*. Gene *ARID4A* plays an important role in regulating Sertoli cells in spermatogenesis [39]. Of special interest, functional haplotypes of this gene were associated with semen quality in Holstein bulls [40]. Gene *TOMM20L* is mostly expressed in testis and regulates different sperm functions, such as motility and viability [41]. Finally, gene *DAAM1* has a fundamental role in cytoskeletal organization in testis and sperm production in rats [42].

The region in BTA13 harbors gene *MYO3*. The protein encoded by *MYO3* belongs to the myosin superfamily and its expressed only in testis. During spermiogenesis, myosins participate in acrosomal formation, nuclear morphogenesis, mitochondrial translocation and spermatid individualization [43]. Finally, in BTA17, we found a significant ROH region with at least two putative genes related to male fertility, *TBX3* and *TBX5*. These T-box genes encode transcription factors that are directly involved in the regulation of different developmental processes. These two transcript factors are highly expressed in testis and prostate, and play key roles in the development of the mammalian reproductive system [44].

## Conclusions

This study evaluated the association between runs of homozygosity and male fertility in dairy cattle. Notably, runs of homozygosity were more prevalent in low-fertility compared to high-fertility Holstein bulls, suggesting that inbreeding and increased homozygosity have a negative impact on dairy bull fertility. Genome-wide mapping of ROH can help to find putative genes affecting bull fertility, and hence, provide a better understanding of the molecular mechanisms underlying male fertility. Here, most of the ROH segments enriched in low-fertility bulls harbor genes directly implicated in testis development, spermatogenesis, and sperm biology. It should be noted that our bull fertility dataset, based on cow field records, is subjected to pre-selection given that only bulls with decent sperm quantity and quality parameters are marketed, and hence, our results should be considered in the context of subfertile bulls in field conditions. Overall, the findings of this study can contribute to the design of mating programs that avoid the production of homozygous offspring which may carry deleterious alleles for male fertility.

## Methods

### Phenotypic and genotypic data

Sire conception rate (SCR) was used as a measured of bull fertility. It represents the US national phenotypic evaluation of service sire fertility, and it is based on confirmed pregnancy records. The statistical model for evaluating dairy bull fertility considers both variables related to the sire (including age of the bull and AI organization), and also variables associated with the cow that receives the unit of semen, including herd-year-season, cow age, parity and milk yield [45, 46].

The SCR records from 11,790 US Holstein bulls were used in this study. The SCR values ranged from − 18.4 to + 11.4%, and the number of breedings per bull ranged from 300 to 136,001. These SCR records were released from August 2008 to April 2018 and are freely available in the Council on Dairy Cattle Breeding (CDCB) website (https://www.uscdcb.com/). Since there are Holstein bulls in the dataset with more than one male fertility evaluation, the most reliable value, i.e. the SCR record with most breedings was used for the analyses [25].

The Cooperative Dairy DNA Repository kindly provided 312 k SNP markers for all the 11,790 bulls with male fertility records. Genetic markers that either mapped to sex chromosomes, had a minor allele frequency ≤ 5% or a call rate ≤ 95% were removed from the genotype file [25]. After this quality control, a total of 267,998 SNP markers remained for subsequent analyses.

### Assessment of runs of homozygosity

We used the PLINK toolset version 1.9 in order to measure segments of consecutive homozygous SNP [47]. ROH were discovered using a sliding window of 100 SNP, allowing one possible heterozygous genotype (to account for potential errors in genotyping and imputation) and one missing SNP per window. Briefly, the algorithm in PLINK takes a window of defined SNP and slide this window across the genome. At each position determines whether this window is homozygous (yes/no). Then, for each SNP, it calculates the proportion of homozygous windows that overlap that position. Here, the size of the window was chosen to avoid detecting ROH segments that are IBS but not IBD [48].

The number of ROH, the average and maximum length in kilobases and SNP was calculated for all the animals for chromosomes 1 to 29. The percentage of homozygous regions for each chromosome was also calculated using either the sum or the maximum length of ROH divided by chromosome length in order to look for differences in autozygosity per chromosome. The association of ROH with SCR was assessed by regressing either the mean, sum or maximum lengths of ROH on the SCR per chromosome and also the entire autosomal genome.

### Association between ROH and dairy bull fertility

The entire population was divided into two subsets with extreme phenotypes: the top and bottom 300 bulls of the SCR distribution were used to investigate differences in homozygosity between high-fertility and low-fertility animals. The average SCR and (standard deviation) for the high-fertility and low-fertility groups were + 3.77% (0.73) and − 6.98% (2.28), respectively. The average and the sum of ROH lengths were compared between fertility groups.

The next step was to identify genomic regions where ROH overlapped across individuals. We defined consensus ROH as segments of overlapping ROH that had a minimum of five SNP. The goal was to determine if the proportion of individuals with overlapping ROH was different in low-fertility vs high-fertility group. For each ROH of interest, a Fisher’s exact test using a 2 × 2 table was performed to determine if there was a statistical difference, i.e., if there was a significant enrichment of this ROH region in the low-fertility group. All significant ROH regions were mapped to the latest bovine refence genome assembly (ARS-UCD1.2) in order to identify candidate genes affecting male fertility. The Ensembl BioMart MartView (https://www.ensembl.org/) was used to retrieve the list of genes within each genomic region of interest.

### Validation of significant ROH

The set of significant ROH regions identified in the previous step was validated in the entire US Holstein bull population (11,790 bulls) using the following linear mixed model:

$$ \mathbf{y}=\mathbf{Xb}+\mathbf{Zu}+\mathbf{e} $$

where **y** is the vector of phenotypic records (SCR values); **b** is the vector of fixed effects including each of the significant ROH regions as binary trait (presence/absence); **X** and **Z** are the design matrices relating SCR records to fixed and random effects, respectively; **u** is the vector of animal genetic effects (breeding values) and **e** is the vector of random residuals. The random effects **u** and **e** were distributed as $\mathbf{u}\sim N\left(0,\mathbf{G}{\sigma}_g^2\right)$ and $\mathbf{e}\sim N\left(0,\mathbf{R}{\sigma}_e^2\right)$, where ${\sigma}_g^2$ and ${\sigma}_e^2$ are the additive genetic and residual variances, respectively, **G** is the additive genomic relationship matrix and **R** is an identity matrix. The association of each ROH region with SCR was evaluated using t-test and those ROH with |t-value| ≥ 2 were declared as significantly associated with male fertility, and therefore were considered as validated. This analysis was performed using the BLUPF90 family programs from Ignacy Misztal and collaborators, University of Georgia.

## Supplementary information

**Additional file 1.** List of overlapping ROH detected across the entire genome.

**Additional file 2.** List of ROH segments enriched in low-fertility bulls.

## Data Availability

The phenotypic data are available at the website of the Council on Dairy Cattle Breeding, section Evaluation Results, Sire Conception Rate (SCR) evaluations (https://queries.uscdcb.com/eval/summary/scr_menu.cfm). The genotypic data are available upon reasonable request to the Cooperative Dairy DNA Repository.

## Abbreviations

ROH: Runs of homozygosity
SCR: Sire conception rate
SNP: Single nucleotide polymorphism
